# Solid brain metastasis mimicking intracerebral hematoma on imaging

**DOI:** 10.1016/j.radcr.2024.05.011

**Published:** 2024-05-31

**Authors:** Satoshi Hori, Shoichi Nagai, Yoshinobu Maeda, Kohtaro Tsumura, Satoshi Kuroda

**Affiliations:** a Department of Neurosurgery, Toyama Red Cross Hospital, Toyama, Japan; b Department of Diagnostic Pathology, Toyama Red Cross Hospital, Toyama, Japan; c Department of Neurosurgery, Graduate School of Medicine and Pharmaceutical Science, University of Toyama, Toyama, Japan

**Keywords:** Hyper dense, Computed tomography, Brain metastasis, Intracerebral hemorrhage, Microscopic hematoma

## Abstract

A 79-year-old woman with a history of resection of the ascending colon cancer presented with conscious disturbance, dysarthria, nausea, and dizziness. Computed tomography (CT) revealed striking high-density lesions in the left cerebellum and left frontal lobe with slight perifocal edema. These lesions were suspected the coexistence of spontaneous cerebellar hemorrhage and frontal lobe metastasis, or multiple brain metastases with massive hematoma. Because of the mass effect of the cerebellar lesion and impaired consciousness, she underwent emergency resection of the cerebellar lesion which was found to be composed of grayish abnormal soft solid tissue and did not include an obvious hematoma mass. The pathological findings were consistent with brain metastasis from colon cancer. This is an impressive rare case of intraoperative solid brain metastasis with a clearly homogenous hyper-dense CT appearance mimicking intracerebral hematoma.

## Introduction

Brain metastasis with adenocarcinoma usually presents as a region of low to moderate attenuation on plain computed tomography (CT). It is well known that calcification, hemorrhage, and the densely packed cell structures of tumors can increase pre-contrast attenuation. Calcification is conspicuous on the CT bone window images. Densely packed cell structures usually produce slightly higher density findings than gray matter. Hemorrhagic brain metastasis is relatively evident in the high-density region [1], which often needs to be discriminated from spontaneous cerebral hemorrhage for diagnosis. Here, we demonstrate an impressive case of intraoperative solid brain metastasis with a clearly homogenous hyper-dense CT appearance mimicking intracerebral hematoma.

## Case presentation

**Fig. 1:**
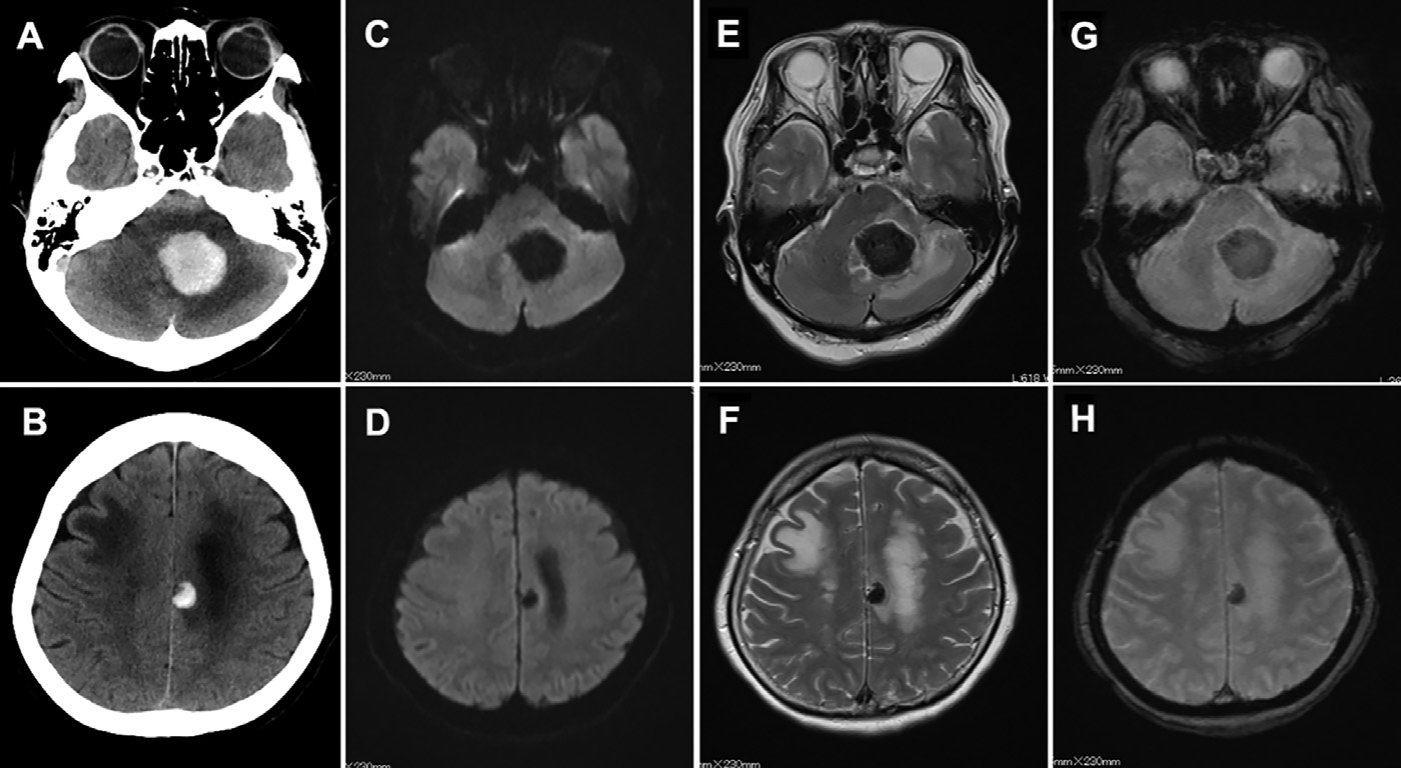
Computed tomography image showing striking high-density lesions in the left cerebellum (A) and left frontal lobe, (B) with slight perifocal edema and edematous change in the right frontal lobe. Diffusion (C, D), T2 (E, F), and T2* (G, H) -weighted magnetic resonance imaging presented homogenous low intensity lesions in the left cerebellum and left frontal lobe, respectively.

**Fig. 2:**
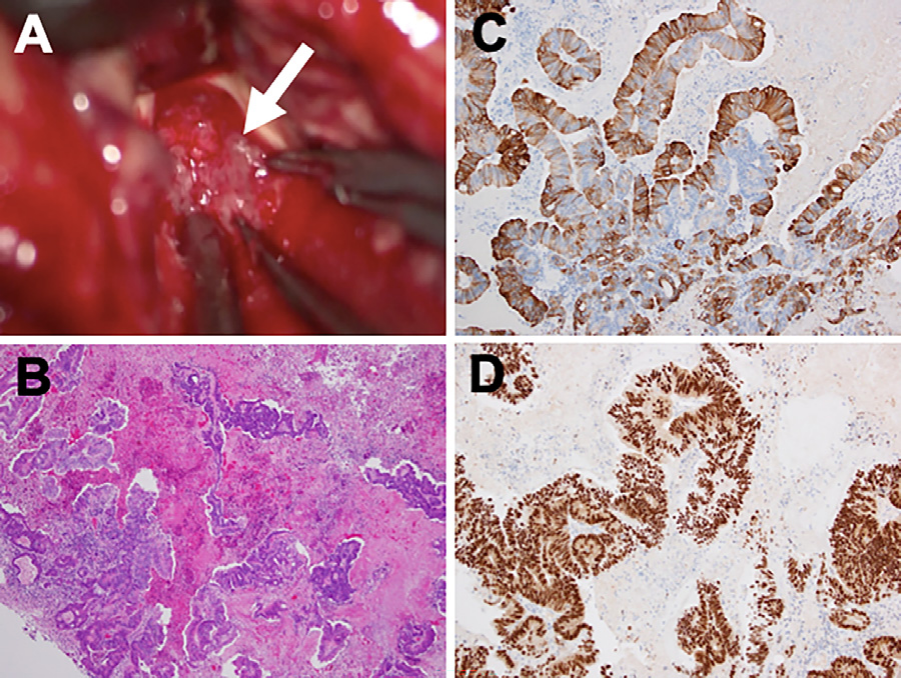
Intraoperative findings demonstrated that the lesion was found to be made up of grayish abnormal soft solid tissue which bled easily (*white arrow*), and obvious hematoma mass could not be confirmed (A). Pathological findings revealed a well differentiated papillotubular adenocarcinoma with hemorrhage in partial area (B). Hematoxylin and eosin staining, original magnification ×200. Immunohistochemical staining with Cytokeratin 20 (C) and CDX-2 (D) revealed positive staining in tumor cells, original magnification ×200.

A 79-year-old woman presented with consciousness disorder in May 2020 and was admitted to our hospital. She had previously undergone resection of adenocarcinoma of the ascending colon cancer in December 2017. Her postoperative course was uneventful, without local recurrence. Although the surgeon recommended aftercare chemotherapy, she refused it and was observed conservatively. On admission to our hospital, her Glasgow Coma Scale score was 13, and neurological examination revealed dysarthria, nausea, and dizziness. Computed tomography (CT) showed striking homogenous high-density lesions in the left cerebellum and frontal lobe with slight perifocal edema and edematous changes in the right frontal lobe (Figs. 1A and B). Lesions in the left cerebellum and frontal lobe presented as homogenous low intensity signals on diffusion-, T2-, and T2*-weighted magnetic resonance (MR) imaging, respectively (Figs. 1C-H). Based on these examinations and the patient's history, we suspected that the coexistence of spontaneous cerebellar hemorrhage and frontal lobe metastasis, or multiple brain metastasis with massive hematoma. Because of the mass effect of the cerebellar lesion and impaired consciousness, the patient was promptly sent to the operating room for emergency resection by posterior fossa craniotomy. Intraoperatively, the lesion was found to be composed of grayish abnormal soft solid tissue, which bled readily and did not include an obvious hematoma mass (Fig. 2A). Pathological findings revealed a well-differentiated papillotubular adenocarcinoma with hemorrhage in partial area (Fig. 2B). Immunohistochemistry was positive for cytokeratin 20 and CDX-2 (Figs. 2C and D). These findings were compatible with the diagnosis of metastatic brain tumor from colon cancer with partial hemorrhage.

## Discussion

This is an impressive rare case of solid brain metastasis mimicking intracerebral hematoma on imaging. Two unique previous reports demonstrated the cases of metastatic brain tumors with clearly high attenuation on CT mimicking hemorrhage, which present as non-hemorrhagic tissue intraoperatively and mucin-producing adenocarcinoma pathologically. It was indicated that lesions containing more mucinous material and less water could produce hyper-dense CT appearance [1,2]. Our case is similar to this situation of which there is an unexpected discrepancy between the preoperative radiological findings and intraoperative findings. However, it differs in terms of pathological findings that our case did not demonstrate the mucin-containing lesion and showed partial hemorrhage within the tumor. On the other hands, Kondziolka et al. described a retrospective review of hemorrhage from brain tumor which is categorized by size into macroscopic or microscopic, which showed that 14.6% of metastatic brain tumors develop intratumoral hemorrhage, macroscopic in 5.4% and microscopic in 9.2% of cases [3].

Based on these findings, our case may be consistent with the metastatic brain tumor with microscopic hemorrhage, which is the possible reason for mimicking intracerebral hemotoma on imaging in spite of intraoperative solid metastatic tumor. However, the radiological findings of brain metastasis with microscopic hemorrhage have not been well understood. It is usual for neurosurgeons to estimate the presence of a gross hematoma as one possibility when the region is shown as a strikingly high attenuation on plain CT. T1-weighted magnetic resonance imaging is considered to one of the useful sequences for diagnosing intracerebral hemorrhage, however, unfortunately, it was not taken in our case. Although the standard for diagnosing metastatic brain tumors is contrast MRI [4], this procedure cannot be performed immediately in all emergent patients. Neurosurgeons should keep in mind that greater awareness of several neuroimaging features is needed for accurate diagnosis and optimal surgical strategies.

## Conclusions

We report an impressive case of intraoperative solid brain metastasis with a clearly homogenous hyper-dense CT appearance mimicking intracerebral hematoma. It may mean the presence of microscopic hemorrhage within the metastatic tumor.

## Patient consent

Informed consent was obtained from the patient for the publication of this case report.
